# Inositol Treatment and ART Outcomes in Women with PCOS

**DOI:** 10.1155/2016/1979654

**Published:** 2016-10-04

**Authors:** Deepika Garg, Reshef Tal

**Affiliations:** ^1^Department of Obstetrics and Gynecology, Maimonides Medical Center, Brooklyn, NY, USA; ^2^Division of Reproductive Endocrinology & Infertility, Department of Obstetrics, Gynecology and Reproductive Sciences, Yale University School of Medicine, New Haven, CT, USA

## Abstract

Polycystic ovary syndrome (PCOS) affects 5–10% of women in reproductive age and is characterized by oligo/amenorrhea, androgen excess, insulin resistance, and typical polycystic ovarian morphology. It is the most common cause of infertility secondary to ovulatory dysfunction. The underlying etiology is still unknown but is believed to be multifactorial. Insulin-sensitizing compounds such as inositol, a B-complex vitamin, and its stereoisomers (myo-inositol and D-chiro-inositol) have been studied as an effective treatment of PCOS. Administration of inositol in PCOS has been shown to improve not only the metabolic and hormonal parameters but also ovarian function and the response to assisted-reproductive technology (ART). Accumulating evidence suggests that it is also capable of improving folliculogenesis and embryo quality and increasing the mature oocyte yield following ovarian stimulation for ART in women with PCOS. In the current review, we collate the evidence and summarize our current knowledge on ovarian stimulation and ART outcomes following inositol treatment in women with PCOS undergoing in vitro fertilization (IVF) and/or intracytoplasmic sperm injection (ICSI).

## 1. Introduction

Polycystic ovarian syndrome (PCOS) is the most common endocrine disorder of women in reproductive age with a prevalence ranging from 5 to 10% [1]. According to the 2003 Rotterdam consensus workshop, it is defined by 2 out of the following 3 criteria: hyperandrogenism, oligo/anovulation, and polycystic ovarian morphology [2]. There is an increased risk of hypertension, cardiovascular disease, impaired glucose tolerance, type II diabetes, and dyslipidemia in women suffering from PCOS. Despite many decades of extensive research, the exact etiology of this disorder remains largely unknown, although it is recognized that ovarian hyperthecosis, increased androgens, insulin resistance, and genetic and environmental factors all play a role in the pathophysiology of this syndrome [3]. It is widely known that insulin resistance (IR) plays an important role in the pathogenesis of PCOS in a large subset of patients [4]. Insulin stimulates ovarian theca cells to produce and secrete androgens both directly and indirectly. Elevated glucose levels in turn inhibit the hepatic synthesis of sex hormone-binding globulin, leading to increased concentration of circulating free androgens [5]. Insulin resistance is a common feature in both overweight and lean women with PCOS independent of their body mass index (BMI) [6]. Approximately 80% of obese women with PCOS and 30–40% of lean women with PCOS have hyperinsulinemia due to IR [7]. As the relationships between IR and PCOS manifestations were established, several insulin-sensitizing compounds such as metformin and thiazolidinediones were demonstrated as effective adjuncts in treating PCOS women [8, 9]. In addition, over the last decade the use of myo-inositol, a natural insulin sensitizer, has been investigated in many studies of PCOS women. The role of myo-inositol in women with PCOS can be attributed to deficiency of metabolites of myo-inositol which are mediators of insulin action [10]. In addition to the beneficial effects of myo-inositol on insulin resistance and other metabolic aspects, it has been shown to improve oocyte maturation, follicular milieu, and outcomes of ART in women with PCOS [11, 12]. In this article, we review the effects of myo-inositol on ovulatory dysfunction in women with PCOS with a particular focus on the accumulating evidence associating myo-inositol with ART outcome in these women. Better understanding of the role of myo-inositol in the reproductive function in PCOS may improve ART outcomes in women with PCOS.

## 2. Methods

A comprehensive literature search was performed using the electronic databases PubMed, Medline, and Google Scholar for relevant articles in the English language until May 2016. The following combinations of search terms were used: “polycystic ovarian syndrome”, “PCOS”, “inositol”, “ovulatory dysfunction”, “hyperandrogenism”, “insulin resistance”, “ovulation induction”, “in vitro fertilization”, “IVF”, “Assisted reproductive technology”, “ART”, “metformin”, and “treatment outcome”. The abstracts identified were reviewed and evaluated by both authors. Any disagreement between the two researchers was resolved with discussion. The reference lists of the selected papers were manually searched in order to identify additional potentially relevant studies.

### 2.1. Inositols: Biochemistry and Sources

Inositols and their derivatives are sugar alcohols which belong to the vitamin B family, representing nine cyclohexane-1,2,3,4,5,6-hexol stereoisomers. They are chemically stable molecules which can be ingested from the diet [13]. Dietary products containing inositol include fruits, mainly cantaloupe and oranges, high bran content cereals, nuts, and beans. Fresh fruits and vegetable contain more inositol when compared with the frozen, salted, or canned products. The average daily intake of myo-inositol from the diet may vary between 225 and 1500 mg/day per 1800 kcal diet [14]. Inositols are not considered an essential nutrient since they can be formed endogenously from glucose [15]. Myo-inositol (MYO) and D-chiro-inositol (DCI) are the two out of nine existing stereoisomers of inositol that are formed after epimerization of the hydroxyl groups of inositol [12, 16–18]. Myo-inositol is the most abundant form of inositol in both nature and mammalian cells, comprising up to 99% of the inositol amount. The remaining 1% is represented by the other stereoisomer DCI. Endogenously, the synthesis of myo-inositol occurs from glucose-6-phosphate in the two-step process which involves conversion of glucose-6-phosphate to myo-inositol 1-phosphate by inositol-3-phosphate synthase enzyme, which is then dephosphorylated to form free myo-inositol by the enzyme inositol monophosphatase [19]. Myo-inositol is converted into DCI by an NAD/NADH epimerase. The activity of this insulin-dependent enzyme strongly influences the intracellular ratio between these two molecules in adipose, hepatic, or muscle cells [20]. Both of these molecules have been used as insulin-sensitizing agents in women with PCOS [21, 22]. Inositol is present in phospholipids, can stimulate lecithin production, serves as an important component of structural lipids, and also controls the fat and lipid metabolism.

### 2.2. Inositol and Reproductive Function

Inositols and their phosphates function as secondary messenger molecules for various cellular signaling pathways including insulin signal transduction, calcium trafficking, lipid metabolism, cytoskeletal protein assembly, cell growth and differentiation, modulation of serotoninergic pathways, oocyte maturation, and fertility [29]. Uptake of free inositol by tissues occurs by a membrane dependent sodium-inositol cotransporter [30]. However, in comparison to DCI, MYO has 10 times greater affinity for this cotransporter [31]. There is substantial evidence linking inositols to reproductive outcomes. Inositol has been demonstrated to play an important role in oocyte maturation and fertilization via its regulation of calcium signaling pathways [32, 33]. Inositol 1,4,5-trisphosphate receptor (IP3R) plays a key role in triggering the Ca^2+^ release during oocyte maturation and fertilization [34, 35]. Myo-inositol levels in blood and follicular fluid have been shown to positively correlate with oocyte quality and pregnancy outcome in humans [32, 36]. Moreover, supplementation of MYO has been suggested to promote meiotic progression of oocytes, producing better quality oocytes and embryos in mice [37, 38]. In humans, administration of MYO to women prior to hormonal stimulation in IVF cycles has been shown to increase oocyte and embryo quality and reduce the dose of FSH and the days required for stimulation [11, 39]. Furthermore, studies on PCOS women have shown that MYO can improve menstrual regularity, insulin resistance, and oocyte quality and maturation [23, 40, 41].

### 2.3. Inositol, Insulin Resistance, and Ovarian Function in PCOS

It is now recognized that insulin resistance has a critical role in the pathogenesis of PCOS. Hyperinsulinemia secondary to insulin resistance is common in a high proportion of PCOS patients and is associated with metabolic morbidities as well as reproductive dysfunction [42]. Insulin resistance is thought to contribute to hyperandrogenism in PCOS by insulin stimulating ovarian theca cells to produce and secrete androgens and by elevated glucose levels inhibiting the hepatic synthesis of sex hormone-binding globulin, leading to increased concentration of circulating free androgens [5]. While the exact cause of insulin resistance in PCOS is unknown, it has been postulated that a defect in the postreceptor transport of glucose and selective resistance to metabolic actions of insulin may be responsible for hyperinsulinemia in these patients [43, 44]. The well-known association between hyperinsulinemia, hyperandrogenism, and ovulatory dysfunction in PCOS formed a basis for treatment with insulin-sensitizing agents such as myo-inositol, metformin, and thiazolidinediones, which have proven effective in improving insulin resistance as well as ovarian functions in these women [45].

Inositol and its stereoisomers are classified as insulin sensitizers and act as a second messenger of insulin signaling pathways [46]. Indeed, the actions of insulin are mediated by two distinct inositol phosphoglycan (IPG) mediators, incorporating either MYO or DCI, which are released by the hydrolysis of glycosyl-phosphatidyl-inositol lipids on the outer side of the cell membrane. IPG in turn activates the key enzymes controlling the oxidative and nonoxidative glucose metabolism, affecting the intracellular metabolic processes. Both MYO- and DCI-containing IPGs decrease insulin resistance [47].

Women with PCOS have reduced serum level and increased urinary loss of DCI [48, 49]. It was demonstrated that DCI urinary clearance was inversely correlated with insulin sensitivity in PCOS women and was a strong and independent predictor of insulin resistance in these women [48, 49]. Following these observations, it has been established that PCOS women have a dysregulation of inositol metabolism [50], providing a mechanistic link between inositol deficiency and insulin resistance in PCOS. Since IPG signaling pathways are involved in insulin-mediated thecal androgen biosynthesis, defective conversion of MYO to DCI in PCOS patients may also contribute to hyperandrogenism [51]. Indeed, administration of DCI at low doses has been shown to decrease insulin resistance and serum androgens and improve ovulatory frequency in PCOS women [16, 52, 53]. However, when administered at higher doses DCI appears to exert negative effects on the ovaries [54]. In fact, subsequent clinical trials performed with DCI doses of 2.4 g/day were unable to confirm previous positive results on PCOS women, suggesting that DCI may paradoxically worsen the ovarian response in these patients despite normalization of the IR parameters [53].

As mentioned earlier, MYO is converted to DCI via an insulin-induced epimerase enzyme in different tissues based on their requirement for each of these molecules [55, 56]. DCI conversion is reduced in muscle tissue of subjects suffering from insulin resistance due to decreased epimerase activity [20, 57, 58]. These studies included muscle and liver which can develop insulin insensitivity. However, in contrast to these tissues, normal ovaries and those of ovulatory PCOS remain insulin sensitive [59, 60]. Moreover, one study demonstrated higher M/C epimerase specific activity in theca cells of PCOS compared to control women and 4-fold higher MYO : DCI ratio in control than in PCOS [61]. Thus, hyperinsulinemia may alter the MYO : DCI ratio and paradoxically increase DCI concentration within the ovary. Decrease in MYO : DCI ratio has also been recently reported in follicular fluid of PCOS women [62]. These observations support the notion that any further increase in DCI is detrimental to ovarian function, explaining the lack of clinical benefit when using DCI in PCOS women and highlighting the importance of maintaining proper MYO : DCI ratio when administering inositols to PCOS women.

In contrast, the benefit of MYO supplementation in PCOS has been demonstrated by several studies. Administration of MYO combined with folic acid 2 g twice a day for 6 months in PCOS patients showed maintenance of normal ovulatory activity in 72%, with singleton pregnancy rate of 40% during the 6-month observation period [23]. In a recent study by Kamenov et al., 50 anovulatory PCOS patients with insulin resistance were given MYO for three spontaneous cycles and ovulation and pregnancy were achieved in 61.7% and 37.9% of women, respectively. In the women who remained anovulatory, MYO was used in combination with clomiphene citrate for three cycles resulting in ovulation and pregnancy rates of 72.2% and 42.6%, respectively. These patients also had reduction in BMI and HOMA index, suggesting the role of MYO-induced amelioration of insulin resistance, in mediating the improvement in ovarian function in women with PCOS [63]. While the above studies lacked a control group, similar beneficial effects on ovarian function were also noted by Artini et al. in their randomized controlled trial of 50 overweight PCOS patients which were divided into two groups; group A was given MYO 2 g plus folic acid 200 mg daily for 12 weeks and group B was given folic acid 200 mg daily. They found significant improvement in hormonal parameters and restoration of menstrual cyclicity in all amenorrheic and oligomenorrheic patients in group A while no changes were noted in group B, suggesting the role of MYO in improving the reproductive axis in PCOS patients [64]. Moreover, administration of both MYO plus DCI has been shown to be effective in achieving better clinical results in PCOS patients in a combination replicating the plasma physiological ratio (40 : 1) by working at systemic and ovary level [62, 65, 66]. The mechanism of the beneficial effects of MYO on ovarian function in the polycystic ovaries could be due to increase in glucose uptake and facilitating FSH signaling that likely improve oocyte quality and better IVF outcome [67].

Another important consideration when evaluating the effects of inositols in PCOS women is the interaction with obesity. Baillrgeon et al. showed that obese PCOS women have diminished release of DCI-IPG in response to insulin elevation compared to normal weight women [50]. Since this study did not include normal weight PCOS women, it is difficult to ascertain whether the observed abnormal DCI-IPG response was related to PCOS, obesity, or a combination of both. However, the investigators noted that reduced insulin-stimulated DCI-IPG response was significantly associated with obesity (BMI, *r* = −0.56, *P* = 0.025), suggesting that obesity plays a role in abnormal inositol metabolism independently of PCOS. In a randomized trial of inositol treatment in PCOS women, Gerli et al. have shown that metabolic risk factor benefits of inositol treatment were not observed in the morbidly obese subgroup of patients, noting an inverse relationship between BMI and treatment efficacy [12]. Recently, Ferrari et al. studied the effects of MYO/DCI supplementation on the maternal metabolic profile in mouse pregnancies complicated by obesity or metabolic syndrome [68]. For their metabolic syndrome model, they used their previously characterized female heterozygous +/− mice lacking endothelial nitric oxide synthase (eNOS), which after feeding with a high-fat diet for 4 weeks develop metabolic-like syndrome phenotype including obesity, glucose intolerance, elevated systolic blood pressure, low high-density lipoprotein, and high insulin. For obesity model, they used wild-type C57BL/6J female mice fed a high-fat diet from weaning for 4 weeks. The pregnant mice were randomized to receive either MYO/DCI (7.2/0.18 mg/mL, resp.) or water as placebo in control group. Pregnant mice with metabolic-like syndrome showed lower serum glucose levels and leptin levels following MYO/DCI treatment as compared to placebo group. In contrast, pregnant mice with obesity alone did not demonstrate improvement in any of the metabolic parameters as compared to placebo group [68]. It was speculated by the study's investigators that MYO/DCI treatment improves glucose tolerance in metabolic-like syndrome pregnant mice but not in the obese mice, possibly involving its specific effects on the nitric oxide pathway. While the above studies suggest that the beneficial effects of inositols may be reduced in obese population, the majority of studies evaluating the effects of inositol treatment on metabolic as well as reproductive function in PCOS women have not specifically addressed the potential interaction of treatment response with BMI. Future studies are needed to better characterize inositol effects as a function BMI and investigate the potential mechanism/s underlying this differential response.

### 2.4. Inositol and ART Outcomes in PCOS

Inositol plays an important role in the follicular microenvironment and affects oocyte maturation and embryo development [24]. Elevated concentration of MYO in the follicular fluid appears to exert a positive effect on follicular maturity and is a marker of good quality oocytes in women with or without PCOS [25, 69]. Recent studies have evaluated the role of inositol in ART outcome in women with PCOS. The data from these studies support the notion that inositol has a beneficial effect on ovarian stimulation and ART outcomes in PCOS patients.

Papaleo et al. investigated the effect of MYO supplementation of 2 g twice a day on ART outcomes in sixty patients with PCOS undergoing ovarian stimulation for intracytoplasmic sperm injection (ICSI) cycles. They found significant reduction in the total number of days of stimulation (11.4 ± 0.9 versus 12.4 ± 1.4, *P* = 0.01), significantly lower peak E2 levels at hCG administration (2,232 ± 510 versus 2,713 ± 595 pg/mL, *P* = 0.02), and reduction in degenerated oocytes (1.0 ± 0.9 versus 1.6 ± 1.0, *P* = 0.01) without compromising oocyte yield in the myo-inositol group in comparison to folic acid alone group [11]. However, no differences were found in fertilization rate, embryo quality, or clinical pregnancy rates between the two groups. The authors also suggested that MYO supplementation may decrease the risk of ovarian hyperstimulation syndrome in PCOS patients [11].

Similar findings were reported by Ciotta et al. in a randomized study in which they evaluated the effects of myo-inositol on oocyte and embryo quality in 34 PCOS patients undergoing IVF/ICSI. Patients in this study were divided into two groups: group A was given myo-inositol (2 g) and folic acid (200 *μ*g) 2 times a day for 3 months, while group B received only folic acid (200 *μ*g). Their results showed lower peak E2 levels at hCG administration, less cycle cancellation, higher number of oocytes retrieved, significantly lower number of immature oocytes, and better quality of embryos with higher number of transferred embryos in group A in comparison to group B [41]. Another recent randomized clinical trial by Unfer et al. aimed at comparing the effects of MYO to DCI on the oocyte and embryo quality in euglycemic patients with PCOS undergoing ovarian stimulation for ICSI. Out of eighty-four women with PCOS in their study, forty-three were given MYO 2 g twice a day and forty-one women were given DCI 0.6 g twice a day. The results showed significantly increased number of mature oocytes, good quality embryos, and total pregnancies in MYO-treated group in comparison to DCI treated group [70]. Similar negative effects of DCI were also noted in a study by Isabella and Raffone who investigated the role of DCI in 54 women diagnosed with PCOS undergoing ICSI. After excluding patients with insulin resistance and/or hyperglycemia, they were divided into 5 groups (10–12 patients/group) with a placebo group and 4 other groups receiving 300, 600, 1200, or 2400 mg DCI daily for 8 weeks. They found significantly increased number of immature oocytes in the three groups that received the higher doses of DCI (*P* < 0.04), with significant reduction in grade I embryos (*P* = 0.004) in DCI supplementation group suggesting the negative effect of DCI on oocyte and embryo quality and worsening ovarian response with increasing DCI dosage in PCOS patients [54]. These data are consistent with the DCI paradox hypothesis, which suggests that in PCOS patients there is depletion of MYO due to accelerated epimerization from MYO to DCI and thus further increase in DCI may be accountable for poor folliculogenesis and oocyte response in these patients [26, 71]. Similar paradoxical findings were noted in PCOS patients undergoing IVF and treated with metformin for 4–8 weeks where metformin reduced the number of oocytes retrieved [27]. The authors suggested that the mechanism behind their results may be increased DCI release in response to metformin [27].

Colazingari et al. studied the role of combined MYO and DCI in comparison to DCI alone in PCOS patients undergoing IVF. They included PCOS patients with BMI less than 28 and FSH less than 10 IU/L undergoing IVF-ET and treated them with MYO combined with DCI in a physiological ratio (1.1 g myo-inositol plus 27.6 mg of D-chiro-inositol) or with DCI alone (500 mg) for 12 weeks. They found reduced number of degenerated oocytes (1.04 ± 1.15 versus 1.82 ± 1.55), better fertilization rate (0.75 ± 0 versus 0.58 ± 0.29) (*P* < 0.05), number of transferred embryos (2.22 ± 0.74 versus 1.67 ± 0.85, *P* < 0.05), and improved embryo quality (0.96 ± 0.83 versus 0.7 ± 0.73, *P* < 0.05) in MYO-DCI treated group in comparison to DCI only treated group [28].

In the largest study to date evaluating the effects of MYO supplementation in PCOS women undergoing IVF, Pacchiarotti et al. randomized 526 PCOS patients into three groups: control (folic acid: 400 mcg, *n* = 195), group A (myo-inositol: 4000 mg, folic acid: 400 mcg, and melatonin: 3 mg daily, *n* = 165), and group B (myo-inositol: 4000 mg and folic acid: 400 mcg daily, *n* = 166). All patients received their treatment from first day of the menstrual cycle until 14 days after embryo transfer. Patients in group A required decreased dose of gonadotropins (group A 2058 ± 233 versus group B 3113 ± 345 versus control group 3657 ± 633, *P* < 0.001) and had enhanced quality of oocytes (group A: 48.2% versus group B 35.0% versus control group 38.2%) and embryos (45.7% in group A versus 30.4% in group B and 25.6% in the control group), suggesting the synergistic effect of MYO and melatonin on improving oocyte and embryo quality [72].

In another study by Rago et al. the combined effect of MYO and *α*-lipoic acid was studied in PCOS patients with normal BMI who had received MYO alone and undergone ICSI previously. They reenrolled 36 PCOS patients who did not achieve pregnancy and 1 patient who had spontaneous abortion and supplemented them with MYO (2 g) and *α*-lipoic acid (800 mg) per day for 3 months. In MYO and *α*-lipoic acid group, significant reduction was noted in immature oocytes (0.2 ± 0.4 versus 1.0 ± 1.5; *P* < 0.001), with improvement in mature oocytes (0.87 ± 0.9% versus 0.81 ± 3.9%, *P* < 0.05) and increase in grade 1 embryos (75.7% versus 57.7%; *P* < 0.05) and higher number of pregnancies achieved (52% versus 33.3%; *P* < 0.01) in comparison to MYO alone group [73]. While this study is limited by lack of a control group, the results suggest that *α*-lipoic acid may enhance the beneficial effects of MYO in PCOS women.  Table 1 summarizes the results of the clinical trials on the effects of inositols on ART outcomes in PCOS women.

In summary, current data on inositol supplementation and ART outcomes in women with PCOS is limited to small randomized clinical trials which suggest beneficial effects of MYO on folliculogenesis with improved oocyte maturation and embryo quality. However, data on the effects of MYO supplementation on implantation, pregnancy, and live birth rates following ART in these women is scarce. Thus, large clinical trials aimed at assessing these important ART outcomes are needed. In addition, further studies are needed to determine the optimal ratio of administered MYO : DCI resulting in maximal beneficial effect.

## 3. Conclusions

In conclusion, myo-inositol is an insulin sensitizer which appears to have beneficial effects on ovarian function and response to ART in women with PCOS. It induces nuclear and cytoplasmic oocyte maturation and promotes embryo development. In contrast, D-chiro-inositol appears to exert opposite and detrimental effects on the ovary. While accumulating evidence suggests that myo-inositol improves the number of mature oocytes retrieved, oocyte quality, and embryo quality in women with PCOS undergoing ART, data on its effects on pregnancy and live birth rates in these women is much more limited. Further research on larger patient populations is needed to determine whether inositol supplementation, possibly in combination with other drugs, could improve clinical pregnancy and live birth rates in PCOS women undergoing ART. It is an affordable, widely available, and easy to administer agent which has the potential of improving the outcomes of fertility treatments in women with PCOS.

## Figures and Tables

**Table 1:** 

Authors	Study design	Study size	Population characteristics	Type of treatment	Mean age (years)	Mean BMI (kg/m^2^)	Main findings
(Papaleo et al., 2009) [11]	Randomized controlled trial	60 women with PCOS Treatment: 30 Placebo: 30	Women with PCOS as defined by oligo/amenorrhea, hyperandrogenism/nemia, and PCO, undergoing ICSI	Treatment: 2 g myo-inositol twice a day plus 400 mg folic acid Placebo: 400 mg folic acid	Treatment: 36.2 Placebo: 35.4	Treatment: 26.7 Placebo: 26.3	Significant reduction in total rFSH units (26 versus 31.7 IU, *P* = 0.016), number of days of stimulation (11.4 versus 12.4, *P* = 0.002), peak E2 level at hCG administration (2,232 versus 2,713 pg/mL, *P* = 0.002), number of germinal vesicles and degenerated oocytes (1.0 ± 0.9 versus 1.6 ± 1.0), and number of cancelled cycles (1 versus 3, *P* = 0.003) in treatment group versus placebo; no significant differences in mature oocytes, fertilization rate, cleavage rate, embryo quality, implantation rate, clinical pregnancy, or miscarriage rate

(Ciotta et al., 2011) [41]	Randomized controlled trial	34 women with PCOS Group A: 17 Group B: 17	Women with PCOS (Rotterdam criteria) undergoing ICSI or IVF	Group A: 2 g of myo-inositol + 200 *μ*g folic acid twice a day × 3 months Group B: 200 *μ*g folic acid twice a day × 3 months	Age < 40 Mean age: not mentioned	Mean BMI: not mentioned	Significantly reduced total rFSH units, peak E2 level at hCG administration, and cancelled cycles (2 versus 5) in group A versus group B. In group A, higher number of mature oocytes, greater number of oocytes retrieved (12 versus 8.5, *P* < 0.05); significantly higher mean number of transferred embryos with greater number of grade I embryos (30 versus 9, *P* < 0.01) in group A. No significant differences in number of fertilized oocytes and total number of biochemical pregnancies between the two groups

(Unfer et al., 2011) [70]	Randomized trial	84 women with PCOS Group A: 43 Group B: 41	Women with PCOS (Rotterdam criteria) undergoing ICSI	Group A: myo-inositol 2 g twice a day for 8 weeks Group B: D-chiro-inositol (DCI) 0.6 g twice a day for 8 weeks	Group A: 35.5 Group B: 36.5	Group A: 24.6 Group B: 25.3	In group A, significant reduction in total rFSH units (1953.6 versus 2360.5 IU, *P* < 0.01), number of stimulation days (11.1 versus 12.7, *P* < 0.01), and peak estradiol levels (2261.2 versus 2740.0 pg/mL; *P* < 0.01); no cycle cancellation in group A versus 4 in group B (*P* = 0.05); no difference between the total number of oocytes retrieved between two groups; in group A, significantly greater number of mature oocytes (8.21 versus 7.08, *P* < 0.05), number of grade 1 embryos (1.64 versus 0.76, *P* < 0.01), number of pregnancies (22 versus 10; *P* < 0.05); no significant differences in clinical pregnancies (15 versus 5, *P* = NS) and spontaneous abortions (4 versus 3; *P* = NS) in group A versus group B, respectively

(Isabella and Raffone 2012) [54]	Randomized controlled trial	54 women with PCOS Placebo: 11 Group A: 10 Group B: 11 Group C: 10 Group D: 12	Women with PCOS (Rotterdam criteria) undergoing ICSI	Placebo group Group A: DCI 300 mg daily Group B: DCI 600 mg daily Group C: DCI 1200 mg daily Group D: DCI 2400 mg daily Treatment given for 8 weeks	Placebo: 36.9 ± 1.5 Group A: 36.8 ± 1.6 Group B: 36.9 ± 1.52 Group C: 36.7 ± 1.57 Group D: 37.0 ± 1.25	Placebo: 24.4 ± 2.8 Group A: 25.2 ± 3.5 Group B: 24.7 ± 3.5 Group C: 25.1 ± 3.1 Group D: 25.6 ± 2.9	Significantly increased FSH dose (IU) administered in the two highest DCI dose groups versus placebo (placebo 2239.7 versus group A 2379.1 versus group B 2305.9 versus group C 2368 versus group D 2983.0); number of stimulation days significantly greater in the 3 higher dose DCI groups versus placebo (placebo 11.4 versus group A 12.1 versus group B 12.5 versus group C 12.9 versus group D 13.8); estradiol levels at hCG administration significantly increased in highest dose DCI group versus placebo (placebo group 1429.69 versus group D 1490.24); no significant differences in number of cycles cancelled or total number of oocytes retrieved; significantly lower number of mature (MII) oocytes in group D compared to placebo group (*P* < 0.001); significantly lower embryo quality in DCI supplemented groups versus placebo (*P* = 0.004)

(Colazingari et al., 2013) [28]	Randomized trial	100 women with PCOS	PCOS (as per Rotterdam criteria) patients with BMI < 28 and FSH < 10 IU/L undergoing IVF-ET	Group A (*n* = 47): MYO 550 mg and DCI 13.8 mg orally twice a day for 12 weeks Group B (*n* = 53): DCI 500 mg orally twice a day for 12 weeks	Not mentioned	Not mentioned	Decreased dose of rFSH (1,569.0 versus 1,899.2 IU; *P* = 0.04) and lower E2 levels before hCG administration (2,185.09 versus 2,519.85, *P* = 0.05) in the MYO group versus DCI group, respectively; the number of oocytes retrieved was higher in the DCI group (8.35 in the MYO-DCI group versus 10.75 in the DCI group); reduced number of degenerated oocytes in MYO-DCI group (age < 35: 1.04 versus 1.82; age > 35: 1.00 versus 1.45); fertilization rate was higher in MYO-DCI treated group (0.75 versus 0.58 in the DCI treated group; *P* < 0.05). Greater number of transferred embryos in group A versus B (2.22 versus 1.67, *P* < 0.05); higher embryo quality in MYO-DCI treated group (0.96 versus 0.7, *P* < 0.05)

Pacchiarotti et al., 2016 [72]	Randomized, controlled double-blind trial	Control (*n* = 195) Group A (*n* = 165) Group B (*n* = 166)	PCOS (Rotterdam criteria), FSH < 12 IU/L, and BMI (20 to 26) undergoing first time ICSI	Control (folic acid 400 mcg) Group A (MYO 4000 mg + folic acid 400 mcg + melatonin 3 mg daily) Group B (MYO 4000 mg + folic acid 400 mcg); given from cycle day 1 until 14 days after ET	Control: 32 ± 3.6 Group A: 31.2 ± 2.1 Group B: 31.5 ± 2.8	Control: 22.8 ± 1.3 Group A: 22.8 ± 1.3 Group B: 23.1 ± 1.7	Less total gonadotropin dose (IU) administered in group A 2058 versus group B 3113 and versus control group 3657 (*P* < 0.001); increased number of mature oocytes reaching MII stage in group A (48.2%) versus group B (35.0%) and control group (38.2%); increased percentage of grade I embryos in group A (45.7%) versus 30.4% in group B and only 25.6% in the control group

Rago et al., 2015 [73]	Prospective study	37 women with PCOS	PCOS patients based on 2 of the Rotterdam criteria (PCO morphology and oligomenorrhea) undergoing IVF	MYO (2 g) and *α*-lipoic acid (800 mg) per day for 3 months in previously MYO-treated group	18–42 years	<24.9	No differences in total dose of FSH administered and duration of stimulation between two groups (1501.9 versus 1498.0 IU; *P* = 0.961, and 11.6 versus 10.8; *P* < 0.05, resp.); in MYO and *α*-lipoic acid group, significant reduction in immature oocytes (0.2 versus 1.0; *P* < 0.001), with improvement in mature oocytes (0.87% versus 0.81%, *P* < 0.05) and increase in grade 1 embryos (75.7% versus 57.7%, *P* < 0.05) in comparison to MYO alone group; no difference in fertilization and cleavage rates between groups; greater number of pregnancies in MYO and *α*-lipoic acid group in comparison to MYO alone group (52% versus 33.3%, *P* < 0.01)

ICSI, intracytoplasmic sperm injection; IVF, in vitro fertilization; ET, embryo transfer; PCOS, polycystic ovary syndrome; PCO, polycystic ovaries; MYO, myo-inositol; DCI, D-chiro-inositol; BMI, body mass index; rFSH, recombinant follicle stimulating hormone; E2, estradiol; hCG, human chorionic gonadotropin; IU, international units; MII, metaphase II.
